# Crizotinib induces autophagy through inhibition of the STAT3 pathway in multiple lung cancer cell lines

**DOI:** 10.18632/oncotarget.5592

**Published:** 2015-09-10

**Authors:** Liangkun You, Jiawei Shou, Danchen Deng, Liming Jiang, Zhao Jing, Junlin Yao, Hongsen Li, Jiansheng Xie, Zhanggui Wang, Qin Pan, Hongming Pan, Wendong Huang, Weidong Han

**Affiliations:** ^1^ Department of Medical Oncology, Sir Run Run Shaw Hospital, School of Medicine, Zhejiang University, Hangzhou, Zhejiang, China; ^2^ Department of Gynaecology and Obstetrics, Sir Run Run Shaw Hospital, School of Medicine, Zhejiang University, Hangzhou, Zhejiang, China; ^3^ Biomedical Research Center, Sir Run Run Shaw Hospital, School of Medicine, Zhejiang University, Hangzhou, Zhejiang, China; ^4^ Division of Molecular Diabetes Research, Department of Diabetes and Metabolic Diseases Research, Beckman Research Institute, City of Hope National Medical Center, Duarte, CA, USA

**Keywords:** crizotinib, autophagy, STAT3, lung cancer

## Abstract

Autophagy is an evolutionarily conserved survival pathway in eukaryote and is frequently upregulated in cancer cells after chemotherapy or targeted therapy. Thus induction of autophagy has emerged as a drug resistance mechanism. In this study, we found that crizotinib induced a high level of autophagy in lung cancer cells through inhibition of STAT3. Ectopic expression of wild-type or constitutive activated STAT3 significantly suppressed the effect of crizotinib on autophagy. Interestingly, crizotinib-mediated inhibition of STAT3 is in a step-wise manner. Firstly it inhibited cytoplasmic STAT3, which leads to the phosphorylation of EIF2A, then inhibited nuclear STAT3, which leads to the downregulation of BCL-2. Cell death induced by crizotinib was greatly enhanced after the inhibition of autophagy by the pharmacological inhibitors or shRNAs against Beclin-1. Moreover, the autophagy inhibitor HCQ significantly augmented the anti-tumor effect of crizotinib in a mouse xenograft model. In conclusion, crizotinib can induce cytoprotective autophagy by suppression of STAT3 in lung cancer cells. Thus, autophagy inhibition represents a promising approach to improve the efficacy of crizotinib in the treatment of targeted lung cancer patients.

## INTRODUCTION

Lung cancer is the leading cause of cancer mortality worldwide. Non-small cell lung cancer (NSCLC) constitutes 80-85% of all lung cancer cases. Most patients have already reached an advanced stage at the time of diagnosis. Chemotherapy is the main therapeutic strategy for patients with metastatic disease. However, even with the emergence of molecular targeted therapy, the overall 5-year survival for patients with NSCLC is still under 20%.[1] Currently, a series of driver mutations has been identified in lung cancer. As an example, the targeting of EGFR and EML4-ALK have led to encouraging results, but acquired resistance generally follows within 1 year. The mechanism of acquired resistance to targeted therapy includes secondary mutations, *MET* amplifications, IGFR activation, *PIK3CA* mutations, and *KRAS* mutations, among others. [2-5] Thus, the investigation of novel targeted strategies as well as of new ways to counter acquired resistance of targeted agents is a logic next step.

Crizotinib is a multitarget tyrosine kinase inhibitor of MET, EML4-ALK and ROS1. It was approved by the US Food and Drug Administration as an initial treatment for locally advanced or metastatic NSCLCs that harbor the EML4-ALK fusion protein[6], and was also recommended for patients with *MET* amplification. MET encodes a transmembrane receptor tyrosine kinase that is activated by hepatocyte growth factor/scatter factor (HGF/SF). Binding of HGF to MET leads to receptor dimerization and transphosphorylation of the tyrosine residues Tyr 1234 and Tyr 1235 of the receptor kinase domain; this initiates downstream signaling pathways including the RAS-ERK-MAPK cascade, the PI3K-AKT-MTOR pathway and the STAT3 signaling pathway. These signaling pathways ultimately lead to increases in cell proliferation, survival and motility.[7] Protein overexpression or gene amplification of MET has been implicated in the oncogenesis of various cancer types, especially lung cancer.[8, 9] Early data in a phase I clinical trial (NCT00585195) have indicated that crizotinib has potent anti-tumor activity in patients with advanced NSCLC with MET amplification.[10] However, acquired drug resistance inevitably occurs in the application of crizotinib just as with other targeted agents. Possible mechanisms of resistance include secondary gate-keeper mutations as well as the activation of signaling pathways that bypass MET signaling.[4, 11, 12]

Autophagy is an evolutionarily conserved catabolic process that sequesters nonessential intracellular components for lysosomal degradation in eukaryotic cells. Autophagy is triggered by a variety of stress stimuli and is widely involved in the pathogenesis of many diseases, especially cancer.[13] When exposed to cellular stress conditions, such as mutations, radiation, chemotherapy or targeted agents, autophagy is activated to promote the survival of tumor cells under these unfavorable conditions.[14] Thus, autophagy has constantly been referred to as a potential pathway of drug resistance.[15] Accumulating evidence indicates that inhibition of autophagy enhances the efficacy of cytotoxic agents such as cisplatin, doxorubicin, as well as that of targeted agents such as sorafenib and cetuximab [16-19]. The most recent reports have shown that the autophagy inhibitor hydroxychloroquine is tolerable and potentially effective in combination with the MTOR-targeted agent temsirolimus [20] and the proteasome inhibitor bortezomib [21] in phase I trials (www.clinicaltrials.gov). In our previous study, we showed that the epidermal growth factor receptor (EGFR) inhibitors gefitinib and erlotinib both induced autophagy in lung cancer cells. The inhibition of autophagy increased the sensitivity of lung cancer cells to EGFR inhibitors, which suggests a novel approach for the enhancement of targeted therapy for lung cancer [22]. Given that autophagy plays an important role in resistance to anti-cancer drugs, we ask whether autophagy can be activated by the multikinase inhibitor crizotinib, thereby impairing the sensitivity of lung cancer cells to its anti-tumor activity.

In the present study, we first demonstrated that crizotinib activated autophagy in lung cancer cells through the inhibition of cytoplasmic as well as nuclear STAT3 signaling. The blockage of autophagy enhanced the anti-tumor activity of crizotinib both *in vitro* and *in vivo*, which suggests a novel and promising strategy for the increase in clinical efficacy of crizotinib in potential targeted patients.

## RESULTS

### Crizotinib induces autophagy in multiple lung cancer cell lines

**Figure 1:**
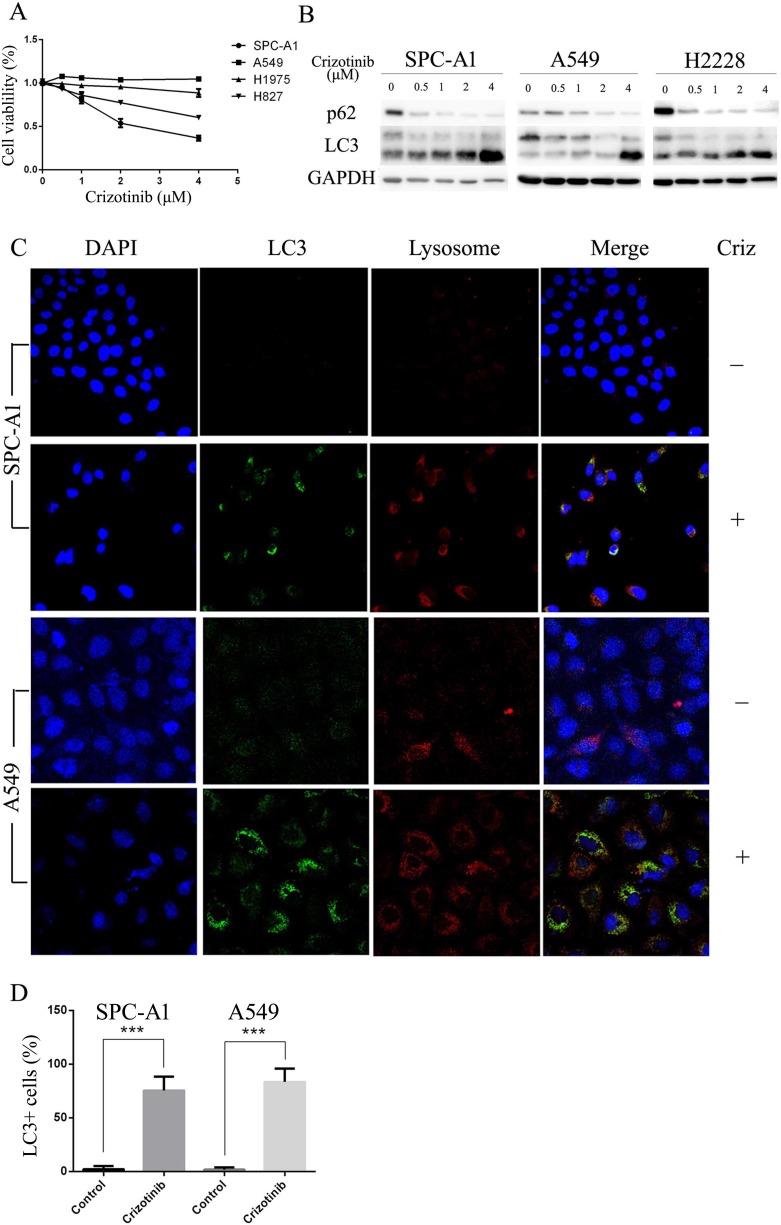
Crizotinib impairs cell viability and activates autophagy in multiple lung cancer cell lines **A**. Cells were incubated with the indicated concentration of crizotinib for 48 h. Cell viability was measured by MTS assay. **B**. Cells were incubated with the indicated concentration of crizotinib for 72 h, and the transition of LC3-I to LC3-II was analyzed by western blot. **C**. Cells were treated with DMSO or 4 μM crizotinib for 72 h before they were labeled with a fluorescent marker and imaged by fluorescence microscopy. Green: FITC-labeled LC3; Red: lyso-tracker-labeled lysosome; Blue: DAPI-labeled nucleus. **D**. The percentage of puncta-positive cells was quantified by automated image acquisition and analysis using a threshold of >5 dots/cell. *** P < 0.001.

**Figure 2:**
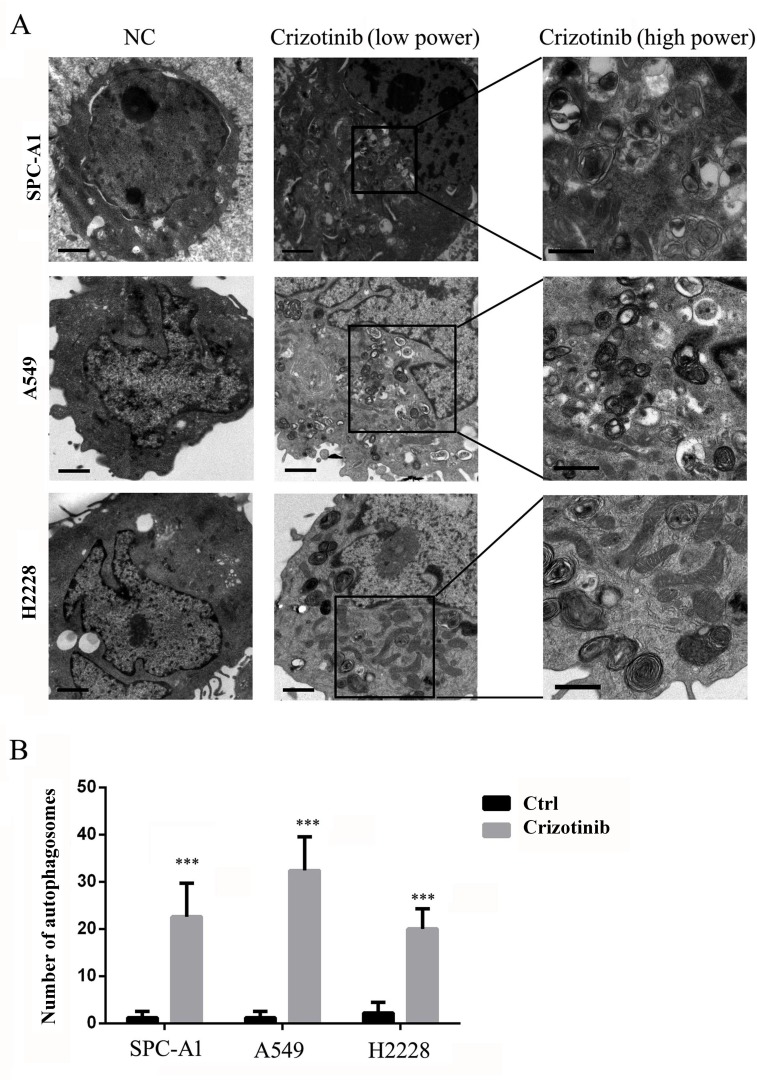
TEM depicts the ultrastructures of autophagosomes in cells treated with crizotinib **A**. Numerous typical autophagosomes with a double-layer membrane appeared in SPC-A1, A549 and H2228 cells that were treated with 4 μM crizotinib for 48 hours. **B**. The number of autophagosomes was calculated by continuous counts within 10 fields under high resolution. Bar = 1 μm. *** P < 0.001.

First, we tested the growth inhibitory properties of crizotinib on a series of lung cancer cells lines including SPC-A1, HCC827, H1975 and A549, and found that although none of these cell lines harbors an EML4-ALK fusion protein, most of them were responsive to crizotinib treatment (Figure 1A). Among these 4 cell lines, SPC-A1 was the most sensitive to crizotinib treatment with an IC50 of approximately 2 μM, while A549 cells, which harbor a KRAS mutation, was the most resistant to crizotinib with an IC50 of over 4 μM. HCC827 and H1975 cells, which have EGFR mutations, exhibited moderate sensitivity. However, all of these cell lines express MET. To evaluate the effect of crizotinib on autophagy, we then examined the occurrence of autophagy in crizotinib-treated lung cancer cells. As shown in Figure 1B, crizotinib induced the lipidation of LC3-II and degradation of p62 in a dose-dependent manner in SPC-A1, A549 and H2228 cells, which indicates the autophagy was activated by crizotinib. To further confirm this observation, the compartmentalization of endogenous LC3-II in cells treated with crizotinib was monitored by indirect immunofluorescence staining. Specific punctate distribution of endogenous LC3-II was observed in crizotinib-treated cells (Figure 1C & Figure S1). After treatment, the percentage of FITC–LC3 positive cells with punctate staining increased to 75.4% and 83.4% in SPC-A1 and A549 cell lines, respectively (Figure 1D). A proportion of LC3-positive cells with punctate staining co-localized with lysosomes, which indicates the formation of autolysosomes. In addition, transmission electron microscopy (TEM) was used to verify the formation of autophagosomes in crizotinib-treated cells. As shown in Figure 2, crizotinib-treated cells exhibited the accumulation of large autophagic vacuoles with a typical double-layer membrane and organelle remnants, whereas only a few vacuoles were observed in control cells.

Collectively, these results demonstrated that crizotinib induces autophagy in lung cancer cells.

### Crizotinib-induced autophagy is mediated through the inhibition of STAT3

**Figure 3:**
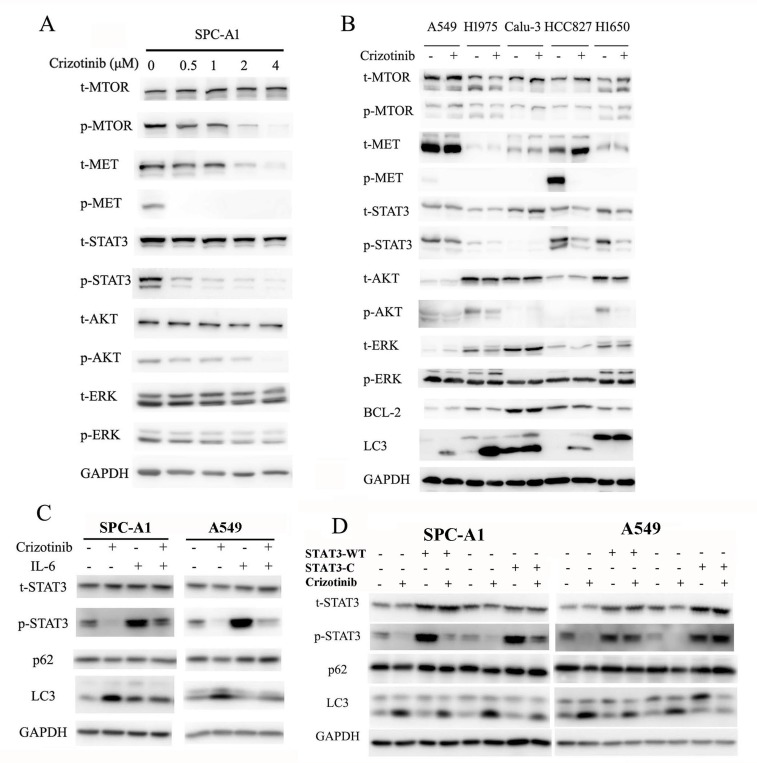
Crizotinib induces autophagy through the inhibition of STAT3 **A**. Immunoblotting for phospho- or total MET, STAT3, AKT, MTOR and ERK in SPC-A1 cells treated with crizotinib for 48 h. **B**. Immunoblotting for phospho- or total MET, STAT3, AKT, MTOR, ERK, BCL-2 and LC3 in cells treated with crizotinib for 48 h. **C**. Immunoblotting for phospho- or total STAT3, p62 and LC3 in SPC-A1 and A549 cells preincubated with 20 ng/ml IL-6 before treated with 4 μM crizotinib for 15 h. **D**. Immunoblotting for phospho- or total STAT3, p62 and LC3 in SPC-A1 and A549 cells transfected with plasmids carrying wild-type STAT3 (STAT3-WT), constitutively activated STAT3 (STAT3-C) or corresponding empty plasmids before treated with crizotinib for 15 h.

Crizotinib was first recognized as a small molecule inhibitor of the MET receptor tyrosine kinase and was approved by the FDA for clinical use in 2011.[23] Three prominent pathways lie downstream of MET (the RAS-MAPK, PI3K-AKT-MTOR and STAT3 signaling pathways), all of which can regulate autophagy.[7, 24] Provided that MET is a direct target of crizotinib, and its downstream pathways are closely involved in the regulation of autophagy, we then hypothesized that crizotinib-induced autophagy is mediated through inhibition of MET. As expected, the phosphorylation of MET was significantly inhibited by crizotinib in SPC-A1 cells, while total MET was unaffected (Figure 3A). The phosphorylation of STAT3, AKT and MTOR in SPC-A1 cells was also repressed by crizotinib in a dose-dependent manner, while the total levels of these proteins were unaffected. However, the phosphorylation of ERK was not affected by crizotinib treatment, which suggests that the ERK pathway is probably not a downstream responder of crizotinib targeting (Figure 3A). We then sought to confirm, by siRNA interference, that the inhibition of MET could induce autophagy. The knockdown of MET in SPC-A1 cells was able to induce the transition of LC3-I to LC3-II (Figure S2A) as well as the punctate staining pattern as observed after immunofluorescence staining (Figure S2B&C). The addition of its ligand HGF could rescue the crizotinib-induced LC3 puncta formation in SPC-A1 cells (Figure S2D). However, we noticed that the direct inhibition of MET did not yield a remarkable autophagic response as shown with crizotinib treatment, as the average percentage of LC3-positive cells with punctate staining was 49% in siMET-transfected SPC-A1 cells (Figure S2C) but was 75.4% in crizotinib-treated cells (Figure 1D). Thus, we tested a series of lung cancer cell lines that reportedly expressed MET. Unexpectedly, although varied expression of total MET was detected in all of the cell lines tested, MET phosphorylation was not commonly present in these cell lines. However, remarkably, in the three cell lines (A549, H1975, H827), autophagy was induced by crizotinib. Not all of these cell lines demonstrated a decreased level of p-MTOR, p-AKT or p-ERK, and the only common downregulated signaling molecule was p-STAT3 (Figure 3B), which suggested crizotinib-induced autophagy was mediated through the inhibition of STAT3. To address this speculation, we used IL-6 to activate STAT3 in lung cancer cells, and found that IL-6 significantly induced phosphorylation of STAT3 and suppressed crizotinib-induced autophagy (Figure 3C). To further address the inhibitory function of IL-6 on autophagy is STAT3-specifically, we then employed plasmids carrying wild-type (STAT3-WT) and constitutively activated STAT3 (STAT3-C). As shown in (Figure 3D), comparing to control empty plasmids, overexpression of total or phosphorylated STAT3 significantly suppressed crizotinib-induced autophagy, reflected by the decrease of LC3 lipidation and increase of p62. Immunofluorescence staining of LC3-II reconfirmed that upregulation of total or phosphorylated STAT3 inhibited crizotinib-induced autophagy, represented by a remarkable decrease of LC3 punctate staining in cells transfected with wild-type or constitutively active STAT3 comparing to cells transfected with control empty plasmids (Figure S3A & S3B). Taken together, these observations indicated that crizotinib-induced autophagy is mediated through the inhibition of STAT3.

**Figure 4:**
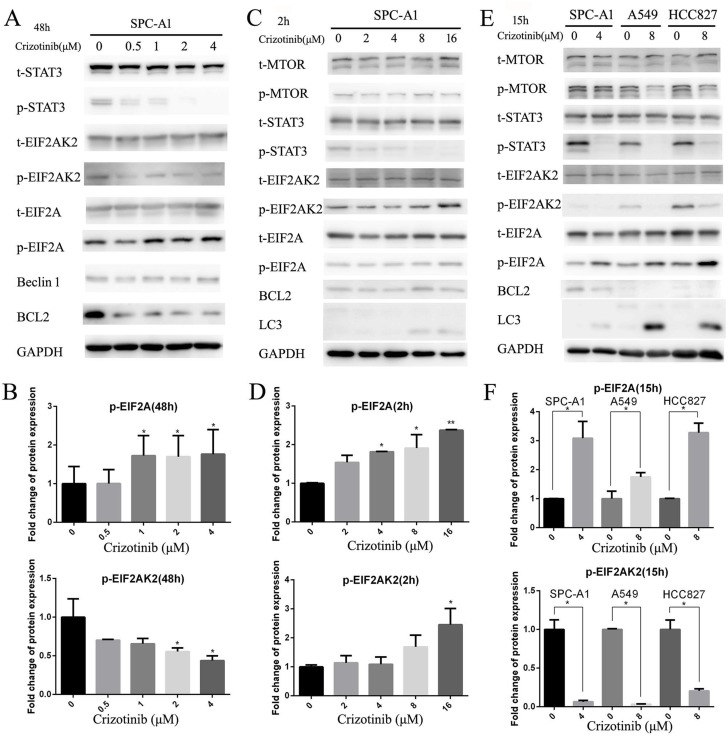
Inhibition of cytoplasmic and nuclear STAT3 pathways both participated in crizotinib-induced autophagy **A**. Immunoblotting for phospho- or total STAT3, EIF2AK2, EIF2A, Beclin-1 and BCL-2 in SPC-A1 cells treated with crizotinib for 48 h. **B**. Quantification of p-EIF2A and EIF2AK2 in panel A. **C**. Immunoblotting for phospho- or total MTOR, EIF2AK2, EIF2A, Beclin-1, BCL-2 and LC3 in SPC-A1 cells treated with crizotinib for 2 h. **D**. Quantification of p-EIF2A and EIF2AK2 in panel C. **E**. Immunoblotting for indicated proteins in cells treated with crizotinib for 15 h. **F**. Quantification of p-EIF2A and EIF2AK2 in panel E. Each column represents three individual experiments. Data are expressed as the mean ± SD. * P<0.05, ** P<0.01.

It has been reported that activated nuclear STAT3 could suppress autophagy by transcriptional activation of BCL-2 family members, which are negative regulators of autophagy.[25] A recent study has provided convincing evidences that the main crosstalk between STAT3 and autophagic pathways occurs in the cytosol. Cytoplasmic STAT3 is able to sequester EIF2AK2, which can phosphorylate EIF2A to promote autophagy.[26] Therefore, we first examined the BCL-2 protein level and the phosphorylation status of EIF2A and EIF2AK2 in SPC-A1 cells after 48 hours of crizotinib treatment (Figure 4A), and discovered that the level of p-EIF2A was only elevated by 1.8-fold compared with the negative control. However, the level of p-EIF2AK2 was decreased 0.4-fold compared with the negative control (Figure 4B). In accordance with the decreased p-STAT3 expression, the level of total BCL-2 was also reduced, which indicates that, in this time period, autophagy was mainly induced by the inhibition of BCL-2 in a STAT3 transcriptionally-dependent manner (Figure 4A). Then, we tested the aforementioned theory once again in a shorter time frame (Figure 4C). This time, it was revealed that the level of p-EIF2AK2 as well as that of p-EIF2A were both significantly increased by 2.5- fold after 2 hours of exposure to crizotinib (Figure 4D), while the level of BCL-2 was unchanged (Figure 4C). Then, again we attempted to confirm these observations in SPC-A1, A549 and HCC827 cells in an intermediate time frame, which is 15 hours of treatment exposure based on the literature [26]; we obtained similar results. It seemed that the phosphorylation of EIF2A and the downregulation of BCL-2 were both responsible for the induction of autophagy at this time point (Figure 4E & 4F).

Taken together, these results demonstrated that crizotinib might induce autophagy in targeted lung cancer cells through the inhibition of the STAT3 pathway in a step-wise manner. This would occur first by the inhibition of cytoplasmic STAT3, which leads to the phosphorylation of EIF2A, then by the inhibition of nuclear STAT3, which leads to the downregulation of BCL-2.

### Inhibition of autophagy potentiates the anti-tumor effects of crizotinib *in vitro*

**Figure 5:**
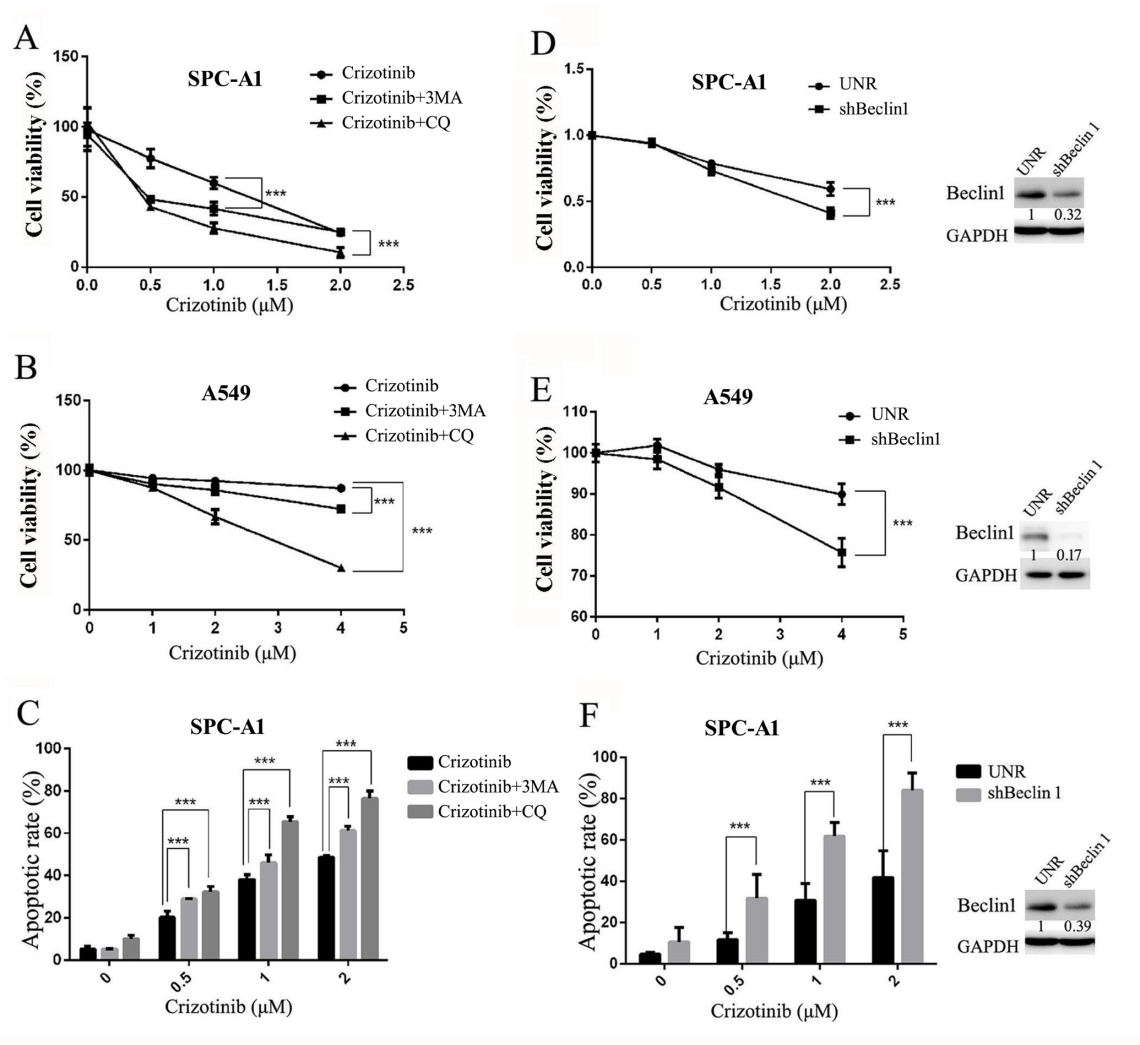
Inhibition of autophagy suppresses cell survival and promotes crizotinib-induced apoptosis *in vitro* **A**. & **B**. Cells were treated with the indicated concentration of crizotinib or DMSO in the presence or absence of CQ (5 μM) or 3-MA (1 mM) for 72 h. Cell viability was measured by MTS assay. **C**. SPC-A1 cells were treated with crizotinib alone or in combination of CQ or 3-MA before staining with annexin V (AV) and propidium iodide (PI), and the apoptotic rates were determined by flow cytometry. **D**. & **E**. Cells were transfected with shRNA against Beclin-1 (shBeclin1) or scramble (UNR) for 24 h and then treated with crizotinib for 48 h. Cell viability was measured by MTS assay. **F**. SPC-A1 cells were transfected with shRNA against Beclin-1 for 24 h and then treated with crizotinib for 48 h before analysis; the apoptotic rates were determined by staining with AV & PI followed by flow cytometry. Each dot or column represents three individual experiments. Data are expressed as the mean ± SD, and analyzed by 2 way ANOVA. *** P<0.001.

Considering that autophagy may function as a stress-activated pro-survival mechanism in cancer cells [27], we hypothesized that the inhibition of autophagy could sensitize targeted lung cancer cells to crizotinib treatment. Two autophagy inhibitors were employed to test the feasibility of this theory: 3-MA, an inhibitor of PIK3C3 that blocks autophagy at the initial stage, and CQ, a lysosomotropic agent, which blocks the fusion of autophagosomes with lysosomes and inhibits autophagy at a later stage [28].** As expected, the addition of either 3-MA or CQ sensitized the growth inhibition induced by crizotinib in SPC-A1 and A549 cells (Figure 5A & 5B). Correspondingly, they were also able to augment the crizotinib-induced apoptosis of SPC-A1 cells (Figure 5C).

To exclude the potential off-target pharmacological effects of drugs that inhibit autophagy, we treated the cells with small hairpin RNAs against Beclin-1, an essential component for the formation of autophagosomes. As shown in Figure 5D & 5E, shRNA against Beclin-1 could efficiently knock down its target gene; it then blocked crizotinib-induced autophagy in SPC-A1 cells as well as in A549 cells. Similar to the action of autophagy inhibitors, shRNA against Beclin-1 significantly enhanced the cytotoxicity of crizotinib in SPC-A1 cells via the inhibition of cell survival and the promotion of apoptosis (Figure 5D, 5E & 5F).

Generally, these data demonstrated that crizotinib-induced autophagy is cytoprotective. Inhibition of autophagy could enhance the growth inhibitory effect of crizotinib, at least partially, through the promotion of apoptosis in targeted lung cancer cells.

### Inhibition of autophagy enhances the anti-tumor effect of crizotinib *in vivo*

**Figure 6:**
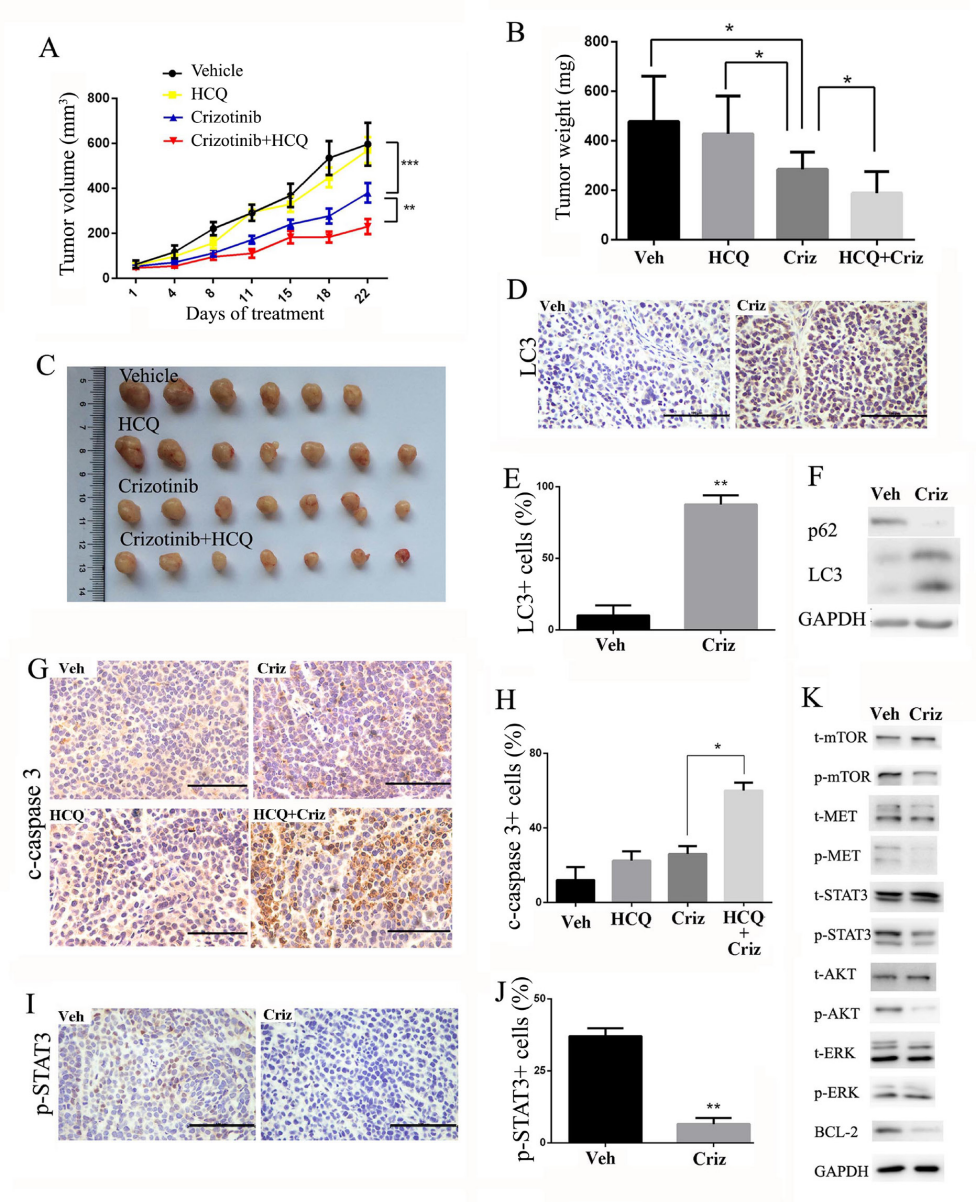
Autophagy inhibition enhances the anti-tumor effect of crizotinib in SPC-A1 xenograft models **A**. Tumor volume in each group. Data are expressed as the mean ± SEM, and were analyzed by 2-way ANOVA. **B**. Tumor weight in each group. Data were expressed as the mean ± SD, and were analyzed by 2 way ANOVA. **C**. Tumors from nude mice in each treatment group. **D**. Immunohistochemical staining for LC3 in vehicle- or crizotinib-treated tumors. **E**. Quantification of LC3-positive cells in xenograft tissue samples. Each column represents samples from five mice. **F**. Tumor lysate was subjected to immunoblotting for LC3 and p62. Each lane represents a mixture of lysate from five mice. **G**. Immunohistochemical staining for cleaved-caspase 3 in paraffin-embedded sections. Quantification of cleaved-caspase 3-positive cells is shown on the right. Each column represents samples from five mice. **H**. Immunohistochemical staining for p-STAT3 in vehicle- or crizotinib-treated tumors. Quantification of p-STAT3-positive cells are shown on the right. Each column represents samples from five mice. **I**. Tumor lysate was subjected to immunoblotting for MET, STAT3, AKT, MTOR, ERK and BCL-2. Each lane represents a mixture of lysates from five mice. Data in E. G. H. are expressed as the mean ± SD. Bar = 100 μm.* P<0.05, ** P<0.01, *** P < 0.001.

To further determine the therapeutic benefit of the inhibition of autophagy in combination with crizotinib, we used a SPC-A1 xenograft model. When the tumors were measurable, the mice were randomly assigned to receive hydroxychloroquine (HCQ), crizotinib or a combination of HCQ and crizotinib. As shown in Figure 6A-6C, HCQ alone had no significant effect on the growth of tumors, and crizotinib alone displayed moderate anti-tumor activity in that it reduced the tumor weight by 40.4%. In contrast, a combination of HCQ with crizotinib further reduced the tumor weight by 20.1% compared with crizotinib alone (Figure 6B). Furthermore, no significant weight loss or hepatic toxicity was observed with the combination treatments (Figure S4A & S4B).

We then investigated the effect of crizotinib on autophagic pathways in xenografted tumors. As shown in Figure 6D & 6E, tumors that were treated with crizotinib displayed an accumulation of LC3-II. In addition, the expression of LC3-II was much higher in the crizotinib-treated group than in the vehicle-treated group. In parallel to the increase in LC3-II, a drastic degradation of the autophagy substrate p62 in tumor tissues was observed after crizotinib treatment. (Figure 6F). An immunohistochemical analysis re-confirmed that the inhibition of autophagy promoted apoptosis, as reflected by positive immunostaining for cleaved-caspase-3 (Figure 6G & 6H). We further noticed that cell proliferation was also downregulated in tumor samples as reflected by PCNA staining (Figure S5), which is probably due to the inhibition of proliferative pathways such as MET signaling. Consistent with the *in vitro* results, crizotinib induced autophagy through the inhibition of expression of p-Met, p-AKT, p-STAT3 and its downstream effector BCL-2. (Figure 6I-6K).

Taken together, these findings suggest that crizotinib induces autophagy in targeted lung cancer cells *in vivo* through the inhibition of the phosphorylation of the STAT3 signaling pathway. Inhibition of autophagy by HCQ can also potently enhance the anti-tumor activity of crizotinib *in vivo*.

## DISCUSSION

In this study, we explored whether crizotinib is able to induce autophagy in the targeted cell lines, and discovered that the treatment induced autophagy in the sensitive SPC-A1 cells, as well as in the resistant A549 cells (Figure 1B, 1C & 1D). Crizotinib treatment can also induce the same effect in EML4-ALK-positive H2228 cells (Figure S1). This observation is consistent with the result of Ji et al's.[11] We observed lipidation of LC3 and degradation of p62 in lung cancer cells after exposure to crizotinib (Figure 1B). Immunofluorescence also showed that the endogenous LC3 punctate staining was increased in crizotinib-treated cells (Figure 1C). A classic autophagy detection method, TEM, also showed that autophagosomes were significantly increased in cells that were treated with crizotinib (Figure 2). These results provided strong evidence that crizotinib activates autophagy in treated lung cancer cells.

MET amplification is present in approximately 4% of lung cancer patients and is related to the acquired resistance to EGFR inhibition as well as ALK inhibition.[2, 3, 5] Aberrant MET activation in cancer is considered a driver oncogene in a subgroup of lung adenocarcinomas.[8, 9] MET is a classic member of the RTK superfamily, which mediates the activation RAS-ERK-MAPK, PI3K-AKT-MTOR and STAT3 pathways, all of which are closely related to autophagy induction.[7, 24] In this study, we first found that the phosphorylation of MET, AKT, MTOR and STAT3 was significantly inhibited in SPC-A1 cells (Figure 3A). In order to confirm that the induction of autophagy by crizotinib in lung cancer cells was the result of MET inhibition, we used small-interference RNA to specifically target *MET*, and revealed that knockdown of *MET* mRNA resulted in the downregulation of MET expression. Therefore, we observed the transition of LC3-I to LC3-II as well as the degradation of p62. Moreover, a marked increase in LC3-II puncta that were colocalized with lysosomes was observed after siMET treatment in SPC-A1 cells. The addition of HGF could reverse the crizotinib-induced punctate LC3 expression (Figure S2). However, we did notice that the induction of autophagy-related markers was far less significant by MET-specific inhibition via siRNA compared with crizotinib treatment. This observation lead us to verify MET expression and downstream signaling in 5 other lung cancer cell lines including A549, H1975, Calu-3, HCC827 and H1650 following crizotinib treatment. Intriguingly, although the expression levels of total MET varied in these cell lines, the baseline level of MET phosphorylation was only detectable in A549 and HCC827 cells, but not in H1975, Calu-3 or H1650 cells. However, in the 3 cell lines (A549, H1975 and HCC827) that autophagy was induced, the downregulation of p-STAT3 was the only factor in common, while the change of p-MET, p-AKT, p-MTOR, or p-ERK was inconsistant (Figure 3B). These findings lead us to focus our effort in the determination of the relationship between crizotinib-induced autophagy and the STAT3 pathway.

In research done by Ji et al.[11], crizotinib induces autophagy in EML4-ALK positive H3122 cells through ALK-dependent downregulation of AKT-MTOR pathway. Whether the treatment alter the STAT3 pathway or not was not mentioned in the original study. However, in our effort to find the common signaling pathway involved in crizotinib treatment, the STAT3 pathway was quite outstanding in all the lung cancer cell lines tested (Figure 3B). Furthermore, we have also examined the phosphorylation status after crizotinib treatment in H2228 cells, a confirmed EML4-ALK positive lung cancer cell line,[8] and discovered the phosphorylation level of STAT3 was downregulated as well (Figure S6). Thus we speculate that inhibition of AKT-MTOR pathway was probably not the main factor in crizotinib-induced autophagy, but the STAT3 pathway. IL-6, plasmids carrying wild-type and constitutively activated STAT3 were then employed to confirm the involvement of STAT3 in crizotinib-induced autophagy. As shown in Figure 3C & 3D and Figure S3, activation of STAT3 by IL6, or overexpression of total or phosphorylated STAT3 significantly suppressed crizotinib-induced autophagy in lung cancer cells.

Intriguingly, we also examined the upstream molecules of STAT3 and discovered that none of JAK1, JAK2 and SRC participated in crizotinib mediated STAT3 inhibition in SPC-A1 cells (Figure S7), which indicates a possible direct inhibition of STAT3 by crizotinib. Nevertheless, this hypothesis remains debatable before further investigation. Moreover, the crizotinib-induced STAT3 inhibition was supported by the research of Hamedani et al.[29], which showed that crizotinib induces apoptosis through the downregulation of STAT3 phosphorylation in NPM-ALK positive anaplastic large cell lymphoma. However genetic inhibition of ALK was not employed in the original work. Therefore, the result of the study should be interpreted with caution, since the downregulation of p-STAT3 could either be the consequence of ALK inhibition or direct crizotinib inhibition.

Studies have shown that STAT3 not only suppresses autophagy via the transcriptional regulation of autophagy-related genes such as BCL-2, [25] but also by the sequestering of cytoplasmic EIF2AK2, which prevents the phosphorylation of EIF2A.[26] Thus, we examined that protein level of BCL-2 as well as the phosphorylation of EIF2AK2 and EIF2A in SPC-A1 cells after 48 hours of crizotinib treatment. As expected, the level of BCL-2 protein was significantly decreased, while EIF2A was increased after treatment with crizotinib; however, the level of p-EIF2AK2 was not increased as presumed, but rather, it was decreased (Figure 4A & 4B). After referred to the original article by Shen et al, [26] we found that the observation of upregulated phosphorylation of EIF2A and EIF2AK2 was not made in the same time frame, but was recorded after 2 hours and 15 hours of autophagy induction, respectively. Therefore, we tested the expression levels of these 2 proteins after 2 hours or 15 hours of crizotinib treatment and obtained very distinct results. After 2 hours of treatment, the phosphorylation of both EIF2AK2 and EIF2A was upregulated, while the level of BCL-2 was unchanged (Figure 4C & 4D). We also noticed that the phosphorylation of MTOR was not decreased in this time-frame. This suggests that the induction of autophagy as evidenced by LC3 transition after 2 hours of crizotinib treatment was not likely due to the transcriptional downregulation of BCL-2 by nuclear STAT3 or loss of MTOR inhibition, but was the outcome of cytoplasmic STAT3 inhibition. We confirmed this hypothesis once again in SPC-A1, A549 and HCC827 cells after 15 hours of crizotinib treatment and obtained similar data (Figure 4E & 4F). This indicates that the increased phosphorylation of EIF2A caused by cytoplasmic STAT3 inhibition as well as by decreased BCL-2, which is mediated by nuclear STAT3 inhibition, might both contribute to the induction of autophagy but that they occur in different time intervals in these lung cancer cell lines. Another interesting observation was also made, in that p-EIF2AK2 was downregulated following prolonged induction of autophagy. This was probably due to post-translational protein modification in the late stage of autophagy flux or severe autophagy degradation, but has not been reported before. This might indicate that p-EIF2AK2 was somehow integrated with or interacted with the autophagosome constructed protein complexes. However, this hypothesis still requires further investigation.

Autophagy has become a potential target for cancer therapy.[15] A number of studies have demonstrated that tyrosine kinase inhibitors (TKI) and antibodies, such as linifanib, [30] imatinib, [31, 32] sorafenib, [18, 33, 34] pazopanib, [35, 36] sunitinib, [36, 37] and cetuximab, [19, 38] among others, can activate autophagy in cancer cells. Our previous study reported that the EGFR tyrosine kinase inhibitors gefitinib and erlotinib activate autophagy in human lung cancer cells.[22] Induction of autophagy in response to these TKIs could be cytoprotective or cytodestructive, which contributes to the anticancer efficacy of these drugs as well as to drug resistance. In our study, we have demonstrated that the inhibition of autophagy sensitizes targeted lung cancer cells to crizotinib treatment *in vitro* and *in vivo*. However, the systemic suppression of autophagy might introduce another problem since the inhibition of autophagy increased the toxicity of chemotherapeutic agents in normal cells. Autophagy in normal cells represents a defense mechanism, and reports have demonstrated that the activation of autophagy protects hepatocytes against chemical-induced hepatotoxicity.[39] Takahashi and colleagues observed that kidney injury was more significant in autophagy-deficient mice compared with wild-type mice after cisplatin administration.[40] Another study also indicated that autophagy was cytoprotective during cisplatin injury of renal proximal tubular cells.[41] In our lung cancer xenograft models, we did observe an insignificant but noticeable decrease in body weight in mice treated with HCQ plus crizotinib (Figure S4A). There was also a report showed that 50 mg/kg crizotinib daily plus 50 mg/kg erlotinib daily could result in severe toxicity in nude mice xenograft models.[42] Thus, it is not clear whether liver injury or chronic kidney injury would occur after crizotinib plus HCQ treatment because the exposure time in this study was relatively short. Therefore, in future studies, we propose a more targeted delivery system in order to increase the benefit of the combined effect of crizotinib and autophagy inhibition.

In summary, we conclude that crizotinib can activate autophagy in lung cancer cells through a STAT3-dependent mechanism in a step-wise manner. In the early stage of crizotinib-induced autophagy, cytoplasmic STAT3 inhibition leads to the phosphorylation of EIF2A, which subsequently induces autophagy. In the later stage of crizotinib-induced autophagy, nuclear STAT3 inhibition leads to the downregulation of BCL-2, which subsequently facilitates a prolonged autophagic response. The inhibition of autophagy by pharmacological or genetic approaches sensitizes targeted lung cancer cells to crizotinib, at least partially, through the promotion of crizotinib-induced apoptosis. Autophagy inhibition represents a promising approach to circumvent intrinsic or acquired resistance to crizotinib in targeted lung cancer cells. Thus, crizotinib combined with autophagy inhibitors could have clinical value in the future.

## MATERIAL AND METHODS

### Reagents and antibodies

The chemicals used in this study were crizotinib (Selleck Chemicals LLC, Houston, TX, USA), chloroquine (CQ), hydroxychloroquine (HCQ) (J&K chemical Ltd, Beijing, China) and 3-methyladenine (3-MA) (Sigma-Aldrich Corporation, St Louis, MO, USA). The primary antibodies used were against microtubule-associated protein 1 light chain 3 (LC-3), p62, Beclin-1, total or phospho-STAT3, AKT, MTOR, ERK, MET, EIF2AK2 and EIF2A and were from Cell Signaling Technology (Boston, MA, USA). The secondary antibodies were HRP-conjugated anti-rabbit IgG, anti-mouse IgG (Cell Signaling Technology) and FITC-conjugated anti-rabbit IgG (Beyotime, Nanjing, China).

### Cell lines and animals

Cells were purchased from the cell bank of the Chinese Academy of Science. The cells were maintained in DMEM (Gibco, Carlsbad, CA, USA) supplemented with 10% fetal bovine serum (Gibco). Crizotinib was dissolved in dimethyl-sulfoxide (DMSO) and was further diluted with medium before use so that the final concentration of DMSO was under 0.1%. Female athymic BALB/c nude mice (Shanghai Institute of Material Medicine, Chinese Academy of Science, China) were maintained in a specific pathogen-free facility and were treated with humane care after approval from the Animal Care and Use Committee of Zhejiang University.

### Proliferation assay

Cell proliferation was determined by MTS assay. The cells were seeded into 96-well plates and were treated with crizotinib, CQ, 3-MA or a combination of these compounds. After treatment, 10 μl MTS (Promega, Madison, WI, USA) was added to each well prior to a 2-h incubation. The absorbance was measured in a model ELX800 Micro Plate Reader (Bio-Tek Instruments, Inc, Winooski, VT, USA) at 490 nm; the proliferation was then calculated.

### Transmission electron microscopy

The treated cells were washed and fixed for 30 min in 2.5% glutaraldehyde. The samples were then treated with 1.5% osmium tetroxide, dehydrated with acetone and embedded in Durcupan resin. Thin sections were poststained with lead citrate and examined in a TECNAI 10 electron microscope (Philips, Eindhoven, Netherlands) at 60 kV.

### Immunofluorescence

The cells, which were seeded at 5 × 10^5^ into six-well plates, were treated with the designated dose of chemicals for 48 h, and were then incubated with Lyso Tracker (Invitrogen, Carlsbad, CA, USA) for 60 min. After this, the cells were washed twice with PBS, followed by fixation in 4% paraformaldehyde and permeabilization with 1% CHAPS buffer (150 mM NaCl, 10 mM HEPES, 1.0% CHAPS) at room temperature for 15 min. Hereafter, the cells were incubated with anti-LC3 (Sigma-Aldrich, L7543) for 2 h at 37°C and with FITC-conjugated anti-rabbit IgG for 1 h at 37°C. Next, the cell nuclei were stained with DAPI (Invitrogen) for 15 min. The samples were examined under a Zeiss LSM 710 fluorescence microscope system (Carl Zeiss Inc, Oberkochen, Germany). Images were processed with ZEN LE software. For quantification of LC3-positive cells, 150-200 cells were randomly selected from the acquired image and were counted. The cells with more than five dots of specific green or orange signals were considered to be LC3-positive.

### RNA interference, plasmids and transfections

Cells were transfected with scrambled or siRNA against *MET* using Hiperfect (Qiagen) according to the manufacturer's protocol. For *MET* RNA interference, two siRNA oligonucleotides that target MET were purchased from GenePharma (Shanghai, China). A nonspecific oligo that is not complementary to any human genes was used as a negative control. The short hairpin RNA (shRNA) sequence that targets Beclin-1 CGACUUGUUCCUUACGGAA was cloned into the pL/shRNA/GFP/F vector purchased from Novobiosci (Shanghai, China). The cells were transduced with pL/shRNA/GFP/F-BECN1 and with vector controls before selection by flow cytometry 24 h later. Then, the cells were maintained in 10 μg/ml blasticidin-containing medium before they were subjected to analysis.

Wild-type STAT3 (pcDNA5/FRT/TO/STAT3-WT), constitutively activated STAT3 (pcDNA3.1/STAT3-C) and their control empty plasmids were kindly provided by Prof. Yu Hua (City of Hope National Medical Center, CA, USA). Cells were transfected with plasmids by Attractene Transfection Reagent (Qiagen) according to the manufacturer's protocol. 48 hours after transfection, the cells were treated with crizotinib and then subjected to analysis.

### Western blot analysis

The cells were lysed and immunoblotted as previously described [43]. Briefly, proteins were resolved by SDS-polyacrylamide gel electrophoresis, transferred to a PVDF membrane (Millipore, Billerica, MA, USA) and were detected by the proper primary and secondary antibodies before visualization with a chemiluminescence kit (Biological Industries, Kibbutz Beth HaEmek, Israel). Visualization was performed with Image Quant LAS-4000 (Fujifilm, Tokyo, Japan) using image Multi-Gauge Software (Fujifilm).

### Apoptosis assay

The cell apoptotic rate was determined by flow cytometric analysis with the fluorescein isothiocyanate (FITC) Annexin V Apoptosis Detection Kit (KeyGEN Biotech, Nanjing, China). Cells were collected by trypsinization, washed twice and resuspended in 1 × binding buffer at a concentration of 1 × 10^6^ cells/ml. Then, 100 μl of cells was mixed with 5 μl of FITC Annexin V and 5 μl PI, and incubated for 15 min. The samples were then sent for analysis by flow cytometry. The results were analyzed with the BD FACSCalibur™ system.

### Human lung cancer xenograft model

To establish SPC-A1 tumors, 5 × 10^6^ cells were inoculated *s.c.* in the flank region of 5- to 6-week-old female athymic BALB/c nude mice. When the diameter of the subcutaneous tumors reached approximately 0.5 centimeter, tumor-bearing animals were randomly assigned to either the vehicle, the crizotinib alone, the HCQ alone or the crizotinib + HCQ group. Crizotinib was suspended in 30% polyethylene glycol, 0.5%Tween 80 and 5% propylene glycol, and administered by oral gavage at the dose of 25 mg/kg/day. HCQ was dissolved in 0.9% NaCl and was administered intraperitoneally daily at a dose of 60 mg/kg/day. The tumor volume was calculated using the formula V = LW^2^/2, where L is the largest diameter and W is the smallest diameter. Mice were sacrificed 24 hours after the last treatment. The tumors were weighed and subjected to western blot analysis or paraffin embedding. Immunohistochemistry was performed on formalin-fixed and paraffin-embedded 4-μm-thick sections of tumor samples using adequate primary antibodies. Images were visualized with a Zeiss LSM 710 fluorescence microscope system (Carl Zeiss Inc). Images were processed with ZEN LE software.

### Ethics statement

The animal study was approved by the institutional animal ethical committee of Zhejiang University with approval No. zju-2013-1-01-066. The methods were performed in accordance with the approved guidelines.

### Statistical analysis

Unless otherwise stated, the data are expressed as the mean ± SD and were analyzed by Student's t test. P** < 0.05 was considered statistically significant.

## SUPPLEMENTARY MATERIAL FIGURES



## References

[1] Allemani C, Weir HK, Carreira H, Harewood R, Spika D, Wang X-S, Bannon F, Ahn JV, Johnson CJ, Bonaventure A, Marcos-Gragera R, Stiller C, Azevedo e Silva G (2015). Global surveillance of cancer survival 1995–2009: analysis of individual data for 25 676 887 patients from 279 population-based registries in 67 countries (CONCORD-2). The Lancet.

[2] Pao W, Chmielecki J (2010). Rational, biologically based treatment of EGFR-mutant non-small-cell lung cancer. Nature reviews Cancer.

[3] Yano S, Takeuchi S, Nakagawa T, Yamada T (2012). Ligand-triggered resistance to molecular targeted drugs in lung cancer: roles of hepatocyte growth factor and epidermal growth factor receptor ligands. Cancer science.

[4] Choi YL, Soda M, Yamashita Y, Ueno T, Takashima J, Nakajima T, Yatabe Y, Takeuchi K, Hamada T, Haruta H, Ishikawa Y, Kimura H, Mitsudomi T (2010). EML4-ALK mutations in lung cancer that confer resistance to ALK inhibitors. The New England journal of medicine.

[5] Kogita A, Togashi Y, Hayashi H, Banno E, Terashima M, De Velasco MA, Sakai K, Fujita Y, Tomida S, Takeyama Y, Okuno K, Nakagawa K, Nishio K (2015). Activated MET acts as a salvage signal after treatment with alectinib, a selective ALK inhibitor, in ALK-positive non-small cell lung cancer. International journal of oncology.

[6] Malik SM, Maher VE, Bijwaard KE, Becker RL, Zhang L, Tang SW, Song P, Liu Q, Marathe A, Gehrke B, Helms W, Hanner D, Justice R (2014). U.S. Food and Drug Administration Approval: Crizotinib for Treatment of Advanced or Metastatic Non-small Cell Lung Cancer that Is Anaplastic Lymphoma Kinase Positive. Clinical cancer research.

[7] Gelsomino F, Facchinetti F, Haspinger ER, Garassino MC, Trusolino L, De Braud F, Tiseo M (2014). Targeting the MET gene for the treatment of non-small-cell lung cancer. Critical reviews in oncology/hematology.

[8] Rikova K, Guo A, Zeng Q, Possemato A, Yu J, Haack H, Nardone J, Lee K, Reeves C, Li Y, Hu Y, Tan Z, Stokes M (2007). Global survey of phosphotyrosine signaling identifies oncogenic kinases in lung cancer. Cell.

[9] (2014). Comprehensive molecular profiling of lung adenocarcinoma. Nature.

[10] Camidge DR, Ou SI, Shapiro G, Otterson GA, Villaruz LC, Villalona-Calero MA, Iafrate AJ, Varella-Garcia M, Dacic S, Cardarella S, Zhao W, Tye L, Stephenson P Efficacy and safety of crizotinib in patients with advanced C-MET amplified non-small cell lung cancer (NSCLC).

[11] Ji C, Zhang L, Cheng Y, Patel R, Wu H, Zhang Y, Wang M, Ji S, Belani CP, Yang JM, Ren X (2014). Induction of autophagy contributes to crizotinib resistance in ALK-positive lung cancer. Cancer biology & therapy.

[12] Yamada T, Takeuchi S, Nakade J, Kita K, Nakagawa T, Nanjo S, Nakamura T, Matsumoto K, Soda M, Mano H, Uenaka T, Yano S (2012). Paracrine receptor activation by microenvironment triggers bypass survival signals and ALK inhibitor resistance in EML4-ALK lung cancer cells. Clinical cancer research : an official journal of the American Association for Cancer Research.

[13] Levine B, Kroemer G (2008). Autophagy in the pathogenesis of disease. Cell.

[14] Pietrocola F, Izzo V, Niso-Santano M, Vacchelli E, Galluzzi L, Maiuri MC, Kroemer G (2013). Regulation of autophagy by stress-responsive transcription factors. Seminars in cancer biology.

[15] Janku F, McConkey DJ, Hong DS, Kurzrock R (2011). Autophagy as a target for anticancer therapy. Nature reviews Clinical oncology.

[16] Xu Y, Yu H, Qin H, Kang J, Yu C, Zhong J, Su J, Li H, Sun L (2012). Inhibition of autophagy enhances cisplatin cytotoxicity through endoplasmic reticulum stress in human cervical cancer cells. Cancer Lett.

[17] Fong MY, Jin S, Rane M, Singh RK, Gupta R, Kakar SS (2012). Withaferin A synergizes the therapeutic effect of doxorubicin through ROS-mediated autophagy in ovarian cancer. PLoS One.

[18] Shimizu S, Takehara T, Hikita H, Kodama T, Tsunematsu H, Miyagi T, Hosui A, Ishida H, Tatsumi T, Kanto T, Hiramatsu N, Fujita N, Yoshimori T (2012). Inhibition of autophagy potentiates the antitumor effect of the multikinase inhibitor sorafenib in hepatocellular carcinoma. Int J Cancer.

[19] Li X, Fan Z (2010). The epidermal growth factor receptor antibody cetuximab induces autophagy in cancer cells by downregulating HIF-1alpha and Bcl-2 and activating the beclin 1/hVps34 complex. Cancer Res.

[20] Rangwala R, Chang YC, Hu J, Algazy KM, Evans TL, Fecher LA, Schuchter LM, Torigian DA, Panosian JT, Troxel AB, Tan KS, Heitjan DF, DeMichele AM (2014). Combined MTOR and autophagy inhibition: Phase I trial of hydroxychloroquine and temsirolimus in patients with advanced solid tumors and melanoma. Autophagy.

[21] Vogl DT, Stadtmauer EA, Tan KS, Heitjan DF, Davis LE, Pontiggia L, Rangwala R, Piao S, Chang YC, Scott EC, Paul TM, Nichols CW, Porter DL (2014). Combined autophagy and proteasome inhibition: A phase 1 trial of hydroxychloroquine and bortezomib in patients with relapsed/refractory myeloma. Autophagy.

[22] Han W, Pan H, Chen Y, Sun J, Wang Y, Li J, Ge W, Feng L, Lin X, Wang X, Jin H (2011). EGFR tyrosine kinase inhibitors activate autophagy as a cytoprotective response in human lung cancer cells. PloS one.

[23] Roskoski R (2013). Anaplastic lymphoma kinase (ALK): structure, oncogenic activation, and pharmacological inhibition. Pharmacological research : the official journal of the Italian Pharmacological Society.

[24] Kroemer G, Marino G, Levine B (2010). Autophagy and the integrated stress response. Molecular cell.

[25] Decuypere JP, Parys JB, Bultynck G (2012). Regulation of the autophagic bcl-2/beclin 1 interaction. Cells.

[26] Shen S, Niso-Santano M, Adjemian S, Takehara T, Malik SA, Minoux H, Souquere S, Marino G, Lachkar S, Senovilla L, Galluzzi L, Kepp O, Pierron G (2012). Cytoplasmic STAT3 represses autophagy by inhibiting PKR activity. Molecular cell.

[27] Levy JM, Thorburn A (2011). Targeting autophagy during cancer therapy to improve clinical outcomes. Pharmacol Ther.

[28] Klionsky DJ, Abdalla FC, Abeliovich H, Abraham RT, Acevedo-Arozena A, Adeli K, Agholme L, Agnello M, Agostinis P, Aguirre-Ghiso JA, Ahn HJ, Ait-Mohamed O, Ait-Si-Ali S (2012). Guidelines for the use and interpretation of assays for monitoring autophagy. Autophagy.

[29] Hamedani FS, Cinar M, Mo Z, Cervania MA, Amin HM, Alkan S (2014). Crizotinib (PF-2341066) induces apoptosis due to downregulation of pSTAT3 and BCL-2 family proteins in NPM-ALK(+) anaplastic large cell lymphoma. Leukemia research.

[30] Pan H, Wang Z, Jiang L, Sui X, You L, Shou J, Jing Z, Xie J, Ge W, Cai X, Huang W, Han W (2014). Autophagy inhibition sensitizes hepatocellular carcinoma to the multikinase inhibitor linifanib. Scientific reports.

[31] Bellodi C, Lidonnici MR, Hamilton A, Helgason GV, Soliera AR, Ronchetti M, Galavotti S, Young KW, Selmi T, Yacobi R, Van Etten RA, Donato N, Hunter A (2009). Targeting autophagy potentiates tyrosine kinase inhibitor-induced cell death in Philadelphia chromosome-positive cells, including primary CML stem cells. The Journal of clinical investigation.

[32] Gupta A, Roy S, Lazar AJ, Wang WL, McAuliffe JC, Reynoso D, McMahon J, Taguchi T, Floris G, Debiec-Rychter M, Schoffski P, Trent JA, Debnath J (2010). Autophagy inhibition and antimalarials promote cell death in gastrointestinal stromal tumor (GIST). Proc Natl Acad Sci U S A.

[33] Shi YH, Ding ZB, Zhou J, Hui B, Shi GM, Ke AW, Wang XY, Dai Z, Peng YF, Gu CY, Qiu SJ, Fan J (2011). Targeting autophagy enhances sorafenib lethality for hepatocellular carcinoma via ER stress-related apoptosis. Autophagy.

[34] Zhai B, Hu F, Jiang X, Xu J, Zhao D, Liu B, Pan S, Dong X, Tan G, Wei Z, Qiao H, Jiang H, Sun X (2014). Inhibition of Akt reverses the acquired resistance to sorafenib by switching protective autophagy to autophagic cell death in hepatocellular carcinoma. Mol Cancer Ther.

[35] Tavallai S, Hamed HA, Grant S, Poklepovic A, Dent P (2014). Pazopanib and HDAC inhibitors interact to kill sarcoma cells. Cancer Biol Ther.

[36] Santoni M, Amantini C, Morelli MB, Liberati S, Farfariello V, Nabissi M, Bonfili L, Eleuteri AM, Mozzicafreddo M, Burattini L, Berardi R, Cascinu S, Santoni G (2013). Pazopanib and sunitinib trigger autophagic and non-autophagic death of bladder tumour cells. Br J Cancer.

[37] Ikeda T, Ishii KA, Saito Y, Miura M, Otagiri A, Kawakami Y, Shimano H, Hara H, Takekoshi K (2013). Inhibition of autophagy enhances sunitinib-induced cytotoxicity in rat pheochromocytoma PC12 cells. J Pharmacol Sci.

[38] Li X, Lu Y, Pan T, Fan Z (2010). Roles of autophagy in cetuximab-mediated cancer therapy against EGFR. Autophagy.

[39] Ni HM, Bockus A, Boggess N, Jaeschke H, Ding WX (2012). Activation of autophagy protects against acetaminophen-induced hepatotoxicity. Hepatology.

[40] Takahashi A, Kimura T, Takabatake Y, Namba T, Kaimori J, Kitamura H, Matsui I, Niimura F, Matsusaka T, Fujita N, Yoshimori T, Isaka Y, Rakugi H (2012). Autophagy guards against cisplatin-induced acute kidney injury. Am J Pathol.

[41] Periyasamy-Thandavan S, Jiang M, Wei Q, Smith R, Yin XM, Dong Z (2008). Autophagy is cytoprotective during cisplatin injury of renal proximal tubular cells. Kidney Int.

[42] Nakade J, Takeuchi S, Nakagawa T, Ishikawa D, Sano T, Nanjo S, Yamada T, Ebi H, Zhao L, Yasumoto K, Matsumoto K, Yonekura K, Yano S (2014). Triple inhibition of EGFR, Met, and VEGF suppresses regrowth of HGF-triggered, erlotinib-resistant lung cancer harboring an EGFR mutation. Journal of thoracic oncology : official publication of the International Association for the Study of Lung Cancer.

[43] Han W, Li L, Qiu S, Lu Q, Pan Q, Gu Y, Luo J, Hu X (2007). Shikonin circumvents cancer drug resistance by induction of a necroptotic death. Mol Cancer Ther.

