# Supplementary material for: Crizotinib induces autophagy through inhibition of the STAT3 pathway in multiple lung cancer cell lines
Source: Oncotarget. 2015 Sep 10;6(37):40268–82. doi: 10.18632/oncotarget.5592 (PMC4741894; doi:10.18632/oncotarget.5592)
Supplement: Supplementary file 1 [file oncotarget-06-40268-s001.pdf]

Crizotinib induces autophagy through inhibition of the STAT3 pathway in multiple lung cancer cell lines

Supplementary Material

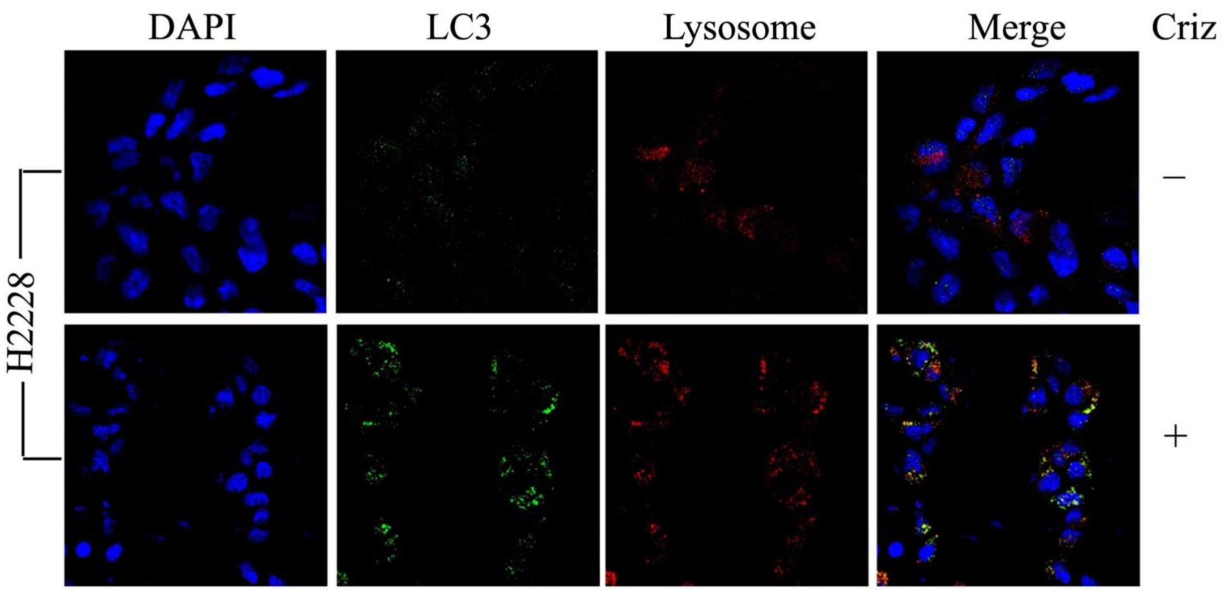

Supplemental Figure 1. Crizotinib induces LC3 puncta formation in H2228 cells. H2228 cells were treated with DMSO or 4 μM crizotinib for 24 h before they were labeled with a fluorescent marker and imaged by fluorescence microscopy. Green: FITC-labeled LC3; Red: lyso-tracker-labeled lysosome; Blue: DAPI-labeled nucleus.

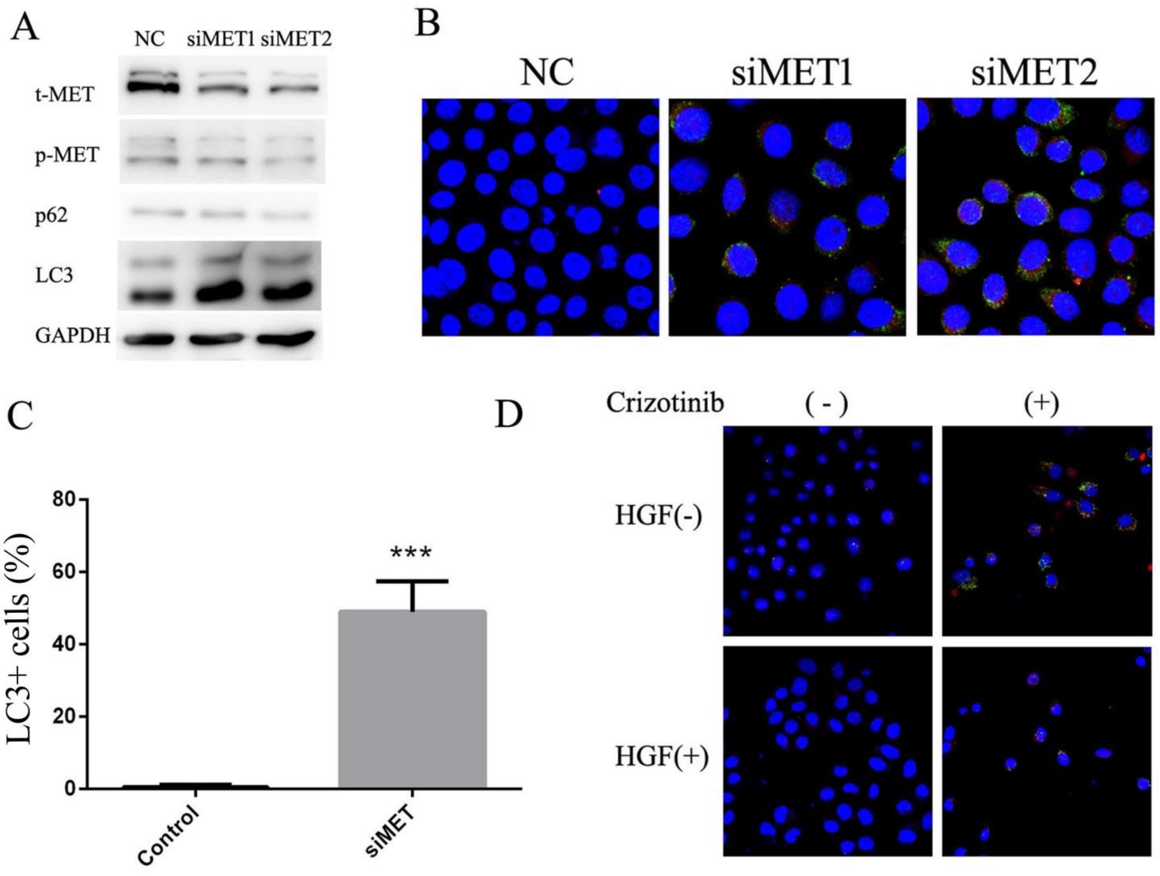

Supplemental Figure 2. Inhibition of MET induces autophagy in SPC-A1 cells. A. Cells were incubated with siMET for 48 h before the phosphorylation of MET, the transition of LC3-I to LC3-II and the degradation of p62 were analyzed by western blot. B. SPC-A1 cells were treated with siMET for 48 h before they were labeled with a fluorescent marker and imaged by fluorescence microscopy. Green: FITC-labeled LC3; Red: lyso-tracker-labeled lysosome; Blue: DAPI-labeled nucleus. C. After siMET transfection, the percentage of puncta-positive cells was quantified by automated image acquisition and analysis using a threshold of >5 dots/cell. D. SPC-A1 cells were pretreated with 50 ng/ml HGF for 2 h and incubated with crizotinib for 48 h before they were labeled with a fluorescent marker and imaged by fluorescence microscopy. Green: FITC-labeled LC3; Red: lyso-tracker-labeled lysosome; Blue: DAPI-labeled nucleus. *** P<0.001.

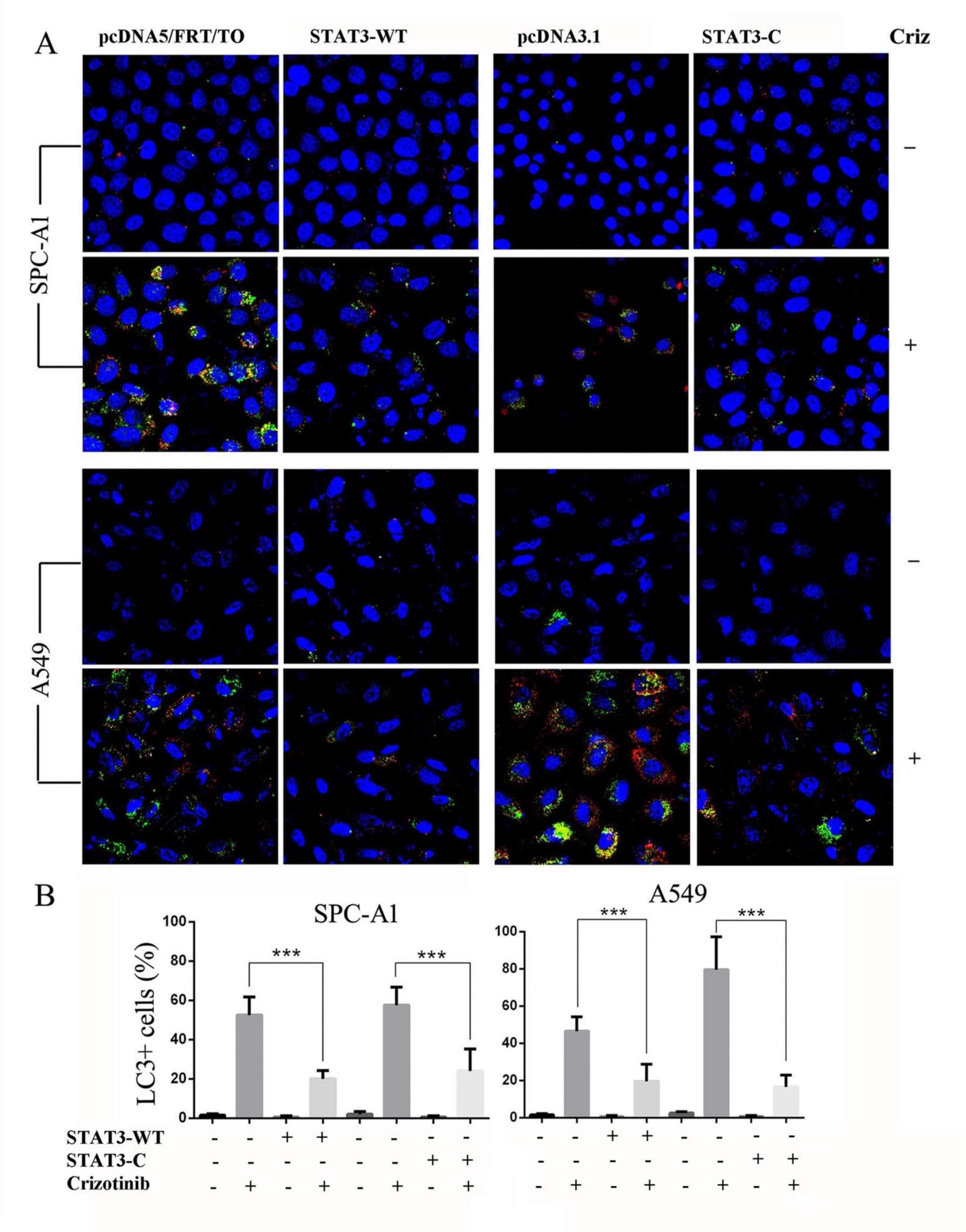

Supplemental Figure 3. Overexpression of total or phosphorylated STAT3 reverses

crizotinib-induced autophagy in SPC-A1 and A549 cells. A. Cells were transfected with plasmids carrying wild-type STAT3, constitutively activated STAT3 or corresponding empty plasmids for 48 hours, and then treated with crizotinib for 15 h. After treatment, cells were labeled with a fluorescent marker and imaged by fluorescence microscopy. Green: FITC-labeled LC3; Red: lyso-tracker-labeled lysosome; Blue: DAPI-labeled nucleus. B. The percentage of puncta-positive cells was quantified by automated image acquisition and analysis using a threshold of >5 dots/cell. *** $P < 0.001$.

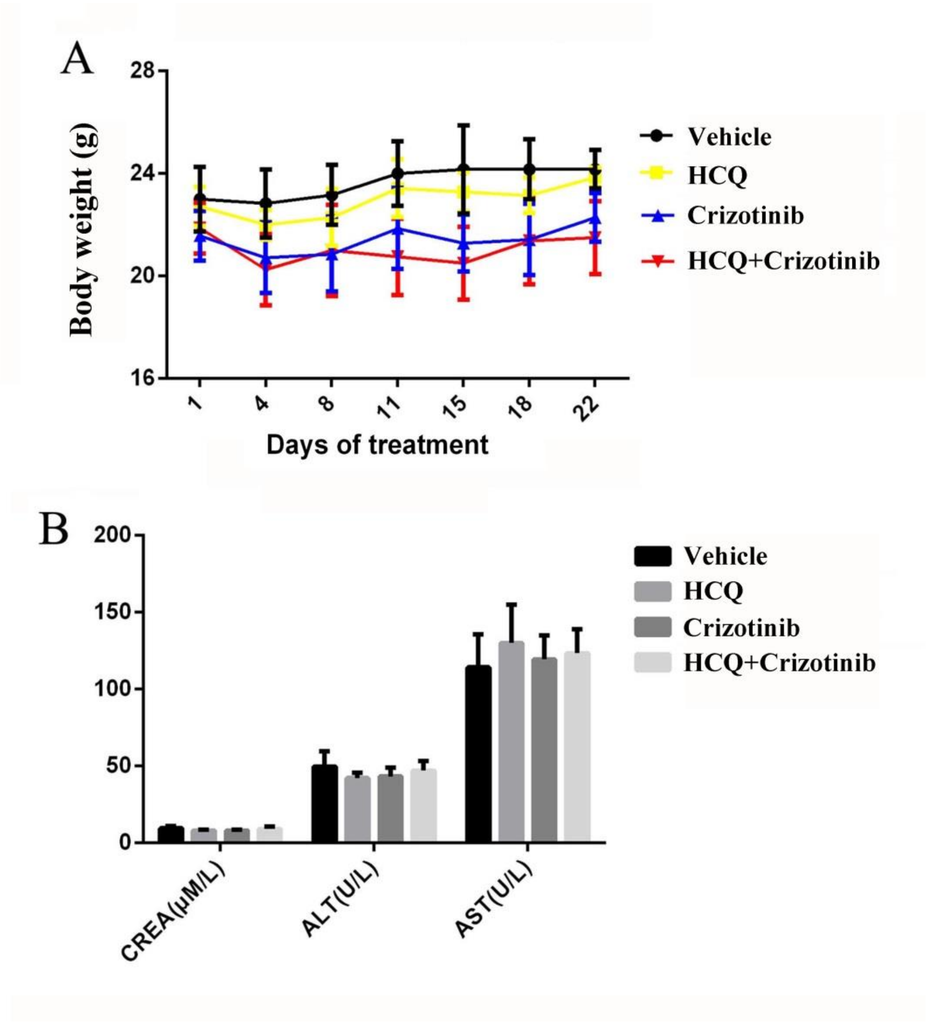

Supplemental Figure 4. Combined treatment of HCQ and crizotinib does not increase significant systematic toxicity in xenograft models. A. The body weight of nude mice in each group. B. The levels of Creatinine (CREA), Alanine transaminase (ALT) and Aspartate transaminase (AST) in each group.

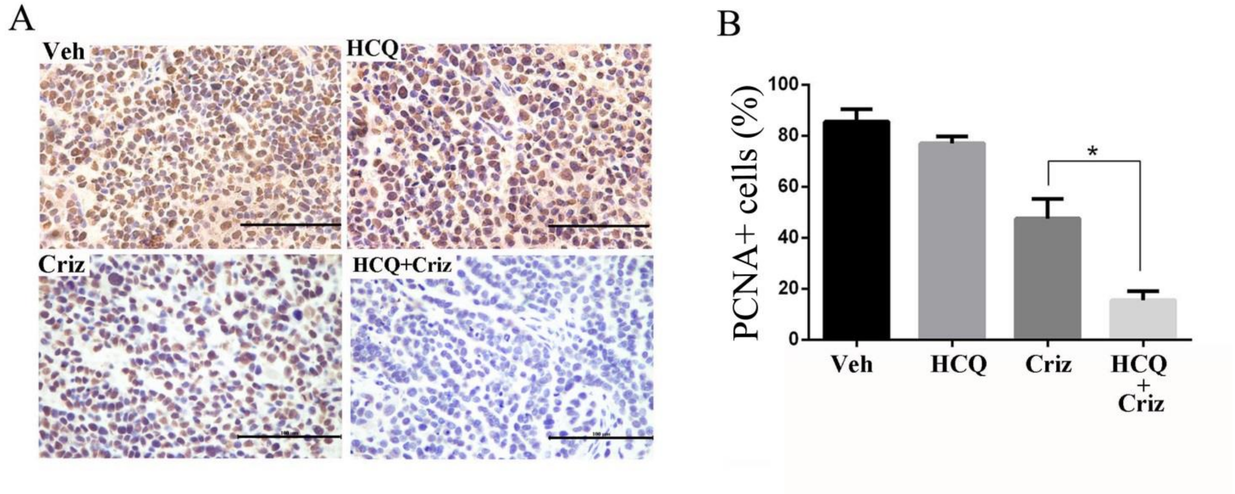

Supplemental Figure 5. Crizotinib inhibits cell proliferation in SPC-A1 xenograft models. A. Immunohistochemical staining of PCNA in paraffin-embedded sections. B. Quantification of PCNA-positive cells in xenograft tumors. Each column represents samples from five mice. Bar = 100 μm. * $P<0.05$.

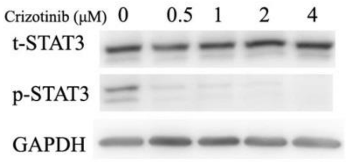

Supplemental Figure 6. Crizotinib downregulates the phosphorylation of STAT3 in H2228 cells. Immunoblotting for phospho- or total STAT3 in H2228 cells treated with indicated concentration of crizotinib for 48 h.

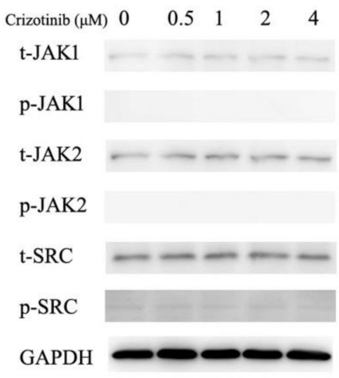

Supplemental Figure 7. JAK1, JAK2 and SRC do not participate in the crizotinib-mediated inhibition of STAT3 in SPC-A1 cells. Immunoblotting for phospho- or total JAK1, JAK2 and SRC in SPC-A1 cells treated with indicated concentration of crizotinib for 48 h.
